# High-Level Mobility of Trans-Tibial Prosthesis Users Wearing Commercial and sPace Energy-Storing Prosthetic Feet

**DOI:** 10.3390/ijerph191912606

**Published:** 2022-10-02

**Authors:** Thanyaporn Rakbangboon, Gary Guerra, Saloottra Kla-arsa, Uthumporn Padungjaroen, Pairat Tangpornprasert, Chanyaphan Virulsri, Kazuhiko Sasaki

**Affiliations:** 1 Sirindhorn School of Prosthetics and Orthotics, Faculty of Medicine, Siriraj Hospital, Mahidol University, Bangkok 10700, Thailand; 2 Department of Exercise and Sport Science, St. Mary’s University, San Antonio, TX 78228, USA; 3 Department of Mechanical Engineering, Faculty of Engineering, Chulalongkorn University, Bangkok 10330, Thailand

**Keywords:** prosthesis, foot, energy storing and return, two-minute walk test, sPace, CHAMP

## Abstract

Outcomes of users provided with a commercial ESR Vari-Flex foot (Össur, Reykjavik, Iceland) and a locally designed sPace foot were investigated. Step activity with users’ own prosthetic foot compared to the sPace foot was explored. Methods: Eleven individuals with unilateral trans-tibial amputation participated and were provided with an sPace and Vari-Flex foot. Ten- and twenty-meter walk tests (10/20MWT) at comfortable and fast walking speeds (CWS/FWS), the two-minute walk test (2-MWT) and Comprehensive High-Level Activity Mobility Predictor (CHAMP) were administered. A subgroup was provided a pedometer to record their steps over a 7-day period in their own foot and later the sPace. Results: The sPace foot performed well in a battery of high-level mobility outcome measures. On CHAMP, participants scored 16.94 ± 5.41 and 16.72 ± 6.09 with the sPace and Vari-Flex feet, respectively. Subgroup testing of step activity showed 4490 ± 3444 steps in users’ own feet and 3115 ± 1967 in the sPace foot, *p* = 0.176. Conclusions: Participants using the sPace foot were capable of performing walking, high-level mobility and activity outcome measures.

## 1. Introduction

Lower limb amputation is a burgeoning global public health issue [1]. The World Health Organization (WHO) estimates that nearly 35–40 million people will need a prosthetic or orthotic device in the coming years [2]. Persons experiencing limb loss are at risk of losing their ability to function independently. Lower limb prostheses offer a means for restoration of quality of life for prosthesis-wearing persons and a reduction in possible comorbidities [3]. Much work has been carried out to enhance the function and comfort of prosthetic technologies. In particular, energy storage and return (ESR) prosthetic feet, although not a recent development, improve gait and satisfaction of users compared to traditional nonenergy-storing feet [4,5,6]. In addition, an ability to traverse across uneven terrain is another hallmark feature of ESR prosthetic feet [7]. Taken together, these features have supplanted the ESR foot as a standard of care for most lower limb prosthesis prescriptions in developed settings.

However, access to these technologies is dependent upon sufficient resources and infrastructure, which is often underdeveloped in developing settings [8]. Although ESR feet are widely available from a plethora of global manufactures, their price precludes them as realistic options for prosthesis users residing in Lower-Middle-Income Countries (LMICs). As such, the World Health Organization (WHO) and UNICEF Global Report on Assistive Technologies call for nations to take necessary steps to insure access to sustainably developed assistive technology [9].

Although ESR feet have been commonplace for decades, in Thailand, they have not been included in the universal coverage healthcare scheme for prosthesis wearers. The Solid-Ankle Cushioned-Heel (SACH) foot remains the standard of care in Thailand. The dynamic motion 1D35 (Otto Bock, Duderstadt, Germany) is offered, but only after absolute medical necessity approval by an expert panel review. The inability of users to obtain affordable ESR feet is hindering prosthetic progress and patient quality of life in resource-limited settings. However, improvements in accessibility to design and testing, as well as manufacturing technologies, has led to the development of a locally designed ESR foot called the sPace [10]. The sPace has received Thailand Food and Drug Administration (FDA) approval and is being registered to the Thai National Health of Security Office (NHSO). The purpose of this study was to investigate the functional outcomes of users provided with a commercial ESR Vari-Flex foot (Össur, Reykjavik, Iceland) and the locally designed sPace foot. A second aim was to examine the free-living physical activity of users wearing their own SACH or a single-axis foot and the sPace foot. Through comparison of these feet, our objective was to explore non-inferiority across the performance of functional outcomes and physical activity for users residing in Thailand.

## 2. Materials and Methods

### 2.1. Participants

This study was approved by the Siriraj Hospital, Faculty of Medicine Institutional Review Board (IRB 593/2560), with each participant signing an informed consent form before participation. Eleven individuals with unilateral trans-tibial amputation, age 49 (range, 27–51), weight 66.5 ± 12.94 and height 164.7 (range, 159.8–169.2), without limb issues or underlying conditions, participated in this study. All participants performed the Amputee Mobility Predictor (AMPro) to make certain their functional activity level was at least a K3 (high-activity) level [11]. Residual limb lengths on all participants were of medium length with a mean time to fit of prosthesis of 3 months. None of the participants had prior experience in wearing an ESR foot and had a minimum of one year of prosthesis experience. Participants wore either a patella-tendon-bearing socket and cuff-strap suspension or total-surface-bearing and suction suspension socket design, and a SACH or single-axis foot (VR rehabpro Co., Ltd., Chachoengsao, Thailand). A prosthetist verified that each participant’s prosthesis was optimized and that their gait was optimal before experimentation.

### 2.2. Prosthetic Foot Provision 

**Figure 1:**
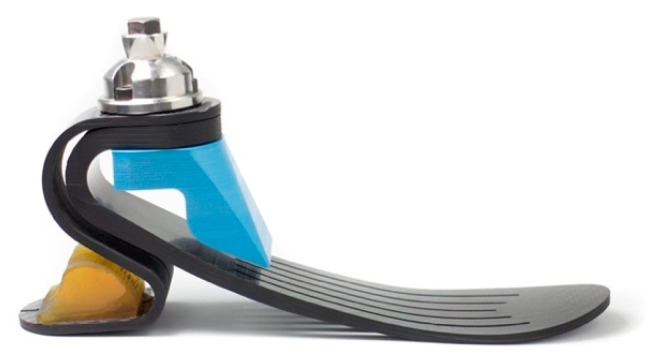
The sPace prosthetic foot.

Participants were randomly allocated to be fit with either an sPace (Department of Mechanical Engineering, Faculty of Engineering, Chulalongkorn University) or a Vari-Flex foot (Össur, Reykjavik, Iceland). The sPace is a carbon-fiber dynamic-response ESR foot with a split heel and a keel that has five slits for simulated eversion and inversion (Figure 1) [10]. The Vari-Flex foot is a dynamic-response carbon-fiber foot that also allows for eversion and inversion via a split keel and heel [12,13]. The prosthetic feet provided to participants were catered to the individual activity level of the participant based on weight and K3 level. Once allocated a prosthetic foot, an investigator fit the prosthesis with a foot on the participant. Thereafter, the participant stood on a L.A.S.A.R Posture (Otto Bock, Duderstadt, Germany) for optimization of prosthesis alignment. Next, an accommodation period in the laboratory was provided for participants. Standardized gait training, which included single-limb balance and cueing to encourage prosthetic toe loading and reduce gait deviations, were encouraged during training [14]. Participants then walked 435 m continuously along a predesignated level indoor walkway at their self-selected walking speed. This accommodation procedure is similar to that used in previous research [15], and is longer than any other protocol used in prosthetic foot research [16]. Participants were given adequate rest time throughout foot accommodation. Once acclimated, participants performed a battery of outcome measurements. This aforementioned process occurred twice, once for each prosthetic foot. 

### 2.3. Outcome Measurement 

A battery of functional outcome measures was administered to all participants while wearing both the sPace as well as the Vari-Flex foot (Figure 1). The two-minute walk test (2-MWT) was administered and required participants to walk as fast as possible along a straight and even 54 m walkway for two minutes [17,18]. A 10 and 20 m (10MWT and 20MWT) walk test was performed at a comfortable walking speed (CWS) and fast walking speed (FWS) [19]. Uneven ten- and twenty-meter walk tests at both CWS and FWS were conducted for participants who could safely ambulate across the uneven surface. A terrasensa relief floor panel (752T1, Otto Bock, Duderstadt, Germany) served as the uneven surface. 

**Figure 2:**
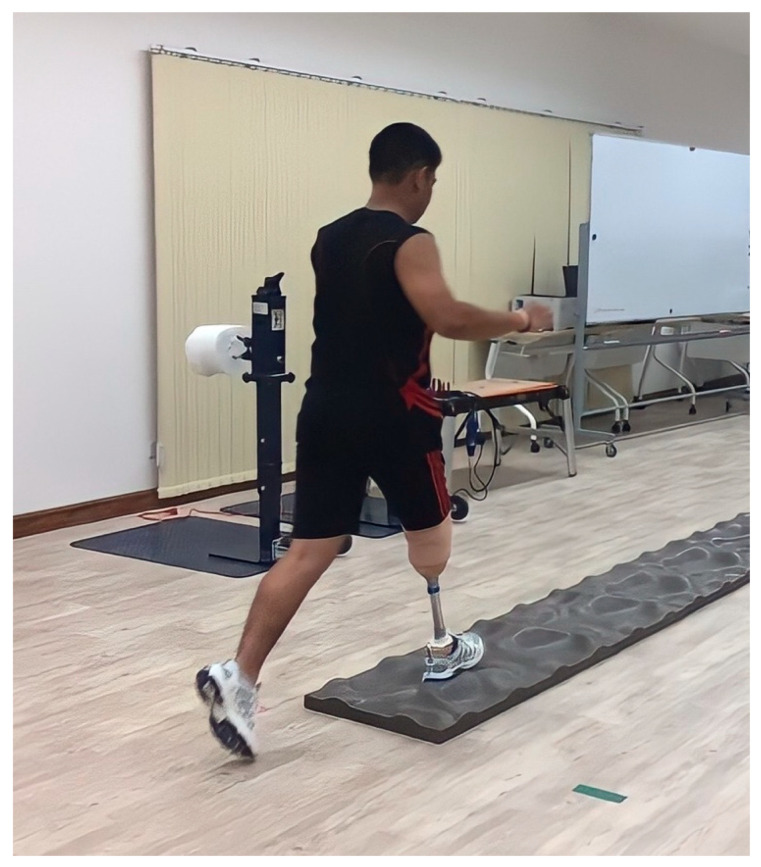
Participant performing uneven terrain outcome measurement.

The Comprehensive High-Level Activity Mobility Predictor (CHAMP) was recently developed as a safe and reliable means of assessing the functional performance of amputees with a high activity level [20,21]. Our CHAMP assessment included the following tests: Single-Limb Stance (SLS) test for assessing balance and walking ability, Edgren Side-Step Test (ESST) for assessing side-step walking, *t*-test for agility, speed and power of movement and lastly the Illinois Agility Test (IAT) for assessing rapid navigation in various directions. The participants’ ability to perform each of the tests determined their high-level mobility and ability to change and shift position quickly. Each participant received instructions for and demonstration of each test prior to testing. Two trials per test occurred with the best score being used for final CHAMP scoring. These outcome measures were performed for each participant on a single day for each prosthetic foot, with sufficient rest time between measurements being provided (Figure 2 shows a photo of the outcome measurement process). Timings for all the above-described outcome measures were measured via stopwatch and recorded to the nearest 10th of a second. 

Lastly, a subset of participants (*n* = 7) participated in a seven-day physical activity assessment. These participants were asked to place a pedometer, Omron HJ-329 (Omron Healthcare, Japan), in their prosthesis side pocket every day for seven days while wearing their own foot (SACH or single-axis), and again in a separate seven-day period for the sPace foot. This activity monitor has good validity and reliability as an activity monitor for prosthesis users [22,23]. Participants returned to the laboratory after their activity assessment period for recording of step counts by an investigator.

### 2.4. Statistical Analysis 

Tests for differences in outcome measures between prosthetic feet were performed using SPSS v27 (IBM, Armonk, NY, USA). A Shapiro–Wilks test was performed to test for normality of data and helped determine appropriate test statistics. To explore non-inferiority between the feet, Mann–Whitney U or *t*-tests were performed depending on the outcome, alpha level *p* = 0.05. To determine non-inferiority margins, minimum detectable change (MDC) scores from the literature were used when possible, and a 10% of the MDC rule was applied [24,25]. Moreover, confidence intervals were used to gauge margins for non-inferiority testing. If MDCs were not available, a test of mean differences was performed using the appropriate statistic. A Pearson correlation was performed to assess the correlation between 2-MWT and CHAMP for both prosthetic feet, *p* = 0.05. Step activity between feet was compared using a Wilcoxon signed-rank test, *p* = 0.05. 

## 3. Results

### 3.1. MWT, 20MWT and 2-MWT 

**Figure 3:**
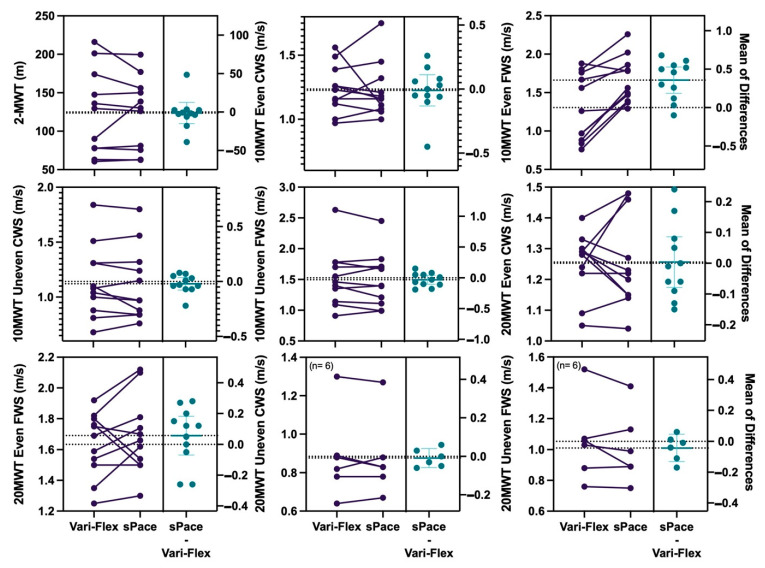
Panel of individual participant differences in outcomes when wearing sPace and Vari-Flex. Note: left panel: participant results between two feet; right panel: mean of differences. 2-MWT: two-minute walk test, 10MWT: ten-meter walk test, 20MWT: twenty-meter walk test, CWS: comfortable walking speed, FWS: fast walking speed, Even; even terrain, Uneven: uneven terrain, m: meters, m/s: meters per second.

Results from even the terrain 10MWT at CWS and FWS show non-inferiority with a mean difference of 0.01 m/s, *p* = 0.015 and a mean difference of 0.40 m/s, *p* = 0.000 respectively. The mean difference for the uneven terrain 10MWT at CWS was 0.03 m/s, *p* = 0.031. However, the uneven terrain 10MWT at FWS did not achieve non-inferiority, at a mean difference of 0.05 m/s, *p* = 0.099. Results from the even terrain 20MWT at CWS and FWS achieved non-inferiority, with mean differences of 0.01 m/s, *p* = 0.013 and mean differences of 0.02 m/s, *p* = 0.03 for CWS and FWS, respectively. The uneven terrain 20MWT at CWS and FWS did not achieve non-inferiority with mean differences of 0.008 (m/s), *p* = 0.05, and a mean difference of 0.04 (m/s), *p* = 0.1. The 2-MWT saw a difference of 1.82 m; however, this result does not satisfy non-inferiority testing, *p* = 0.33. Figure 3 provides a panel overview of differences seen in participants wearing the two study feet (Table 1). 

### 3.2. CHAMP 

**Figure 4:**
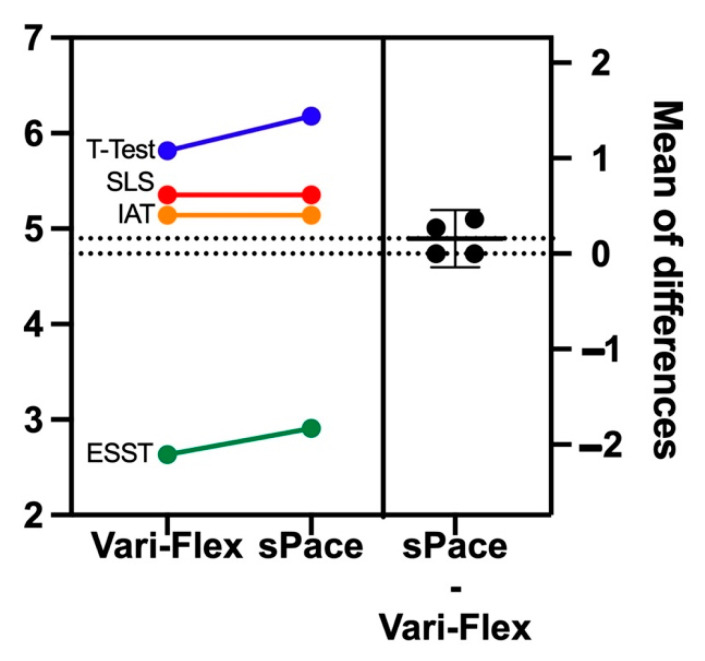
Mean differences of participants wearing sPace and Vari-Flex on CHAMP. Note: left panel is individual test differences for all participants, and right panel is mean differences between feet. CHAMP: Comprehensive High-Level Activity Mobility Predictor, SLS: Single-Limb Stance Test, IAT: Illinois Agility Test, ESST: Edgren Side-Step Test. Individual tests were converted to a test score according to CHAMP guidelines.

**Figure 5:**
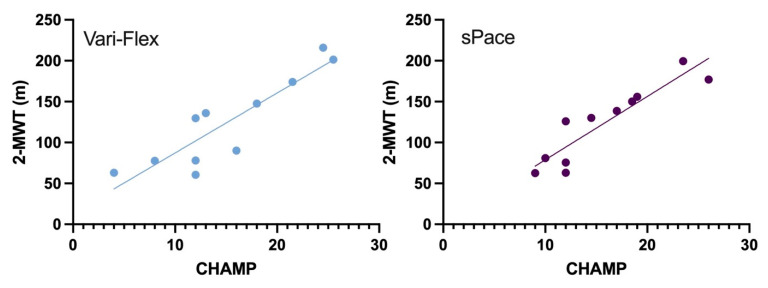
Pearson correlation coefficient between 2-MWT and CHAMP for sPace and Vari-Flex. Note: 2-MWT: two-minute walk test, CHAMP: Comprehensive High-Level Activity Mobility Predictor. The 2MWT units are in meters, and CHAMP units are summed scores of battery of outcome measures.

We report individual test results to show differences between the two prosthetic feet and non-inferiority results. ESST differences showed non-inferiority between feet with a mean difference of 0.11, *p* = 0.049. The IAT did not achieve non-inferiority with a mean difference of 0.00, *p* = 0.097. Likewise, non-inferiority was not seen for the *t*-test, with a mean difference 0.12, *p* = 0.12. The SLS did not achieve non-inferiority with a mean difference of 0.00, *p* = 0.18. Figure 4 illustrates differences observed between the two prosthetic feet in study participants. Pearson correlation coefficient results indicate an *r* = 0.89 *p* = 0.0002 for Vari-Flex on 2-MWT and CHAMP, and *r* = 0.91 *p* ≤ 0.0001 with the sPace (Figure 5).

### 3.3. Step Activity 

**Figure 6:**
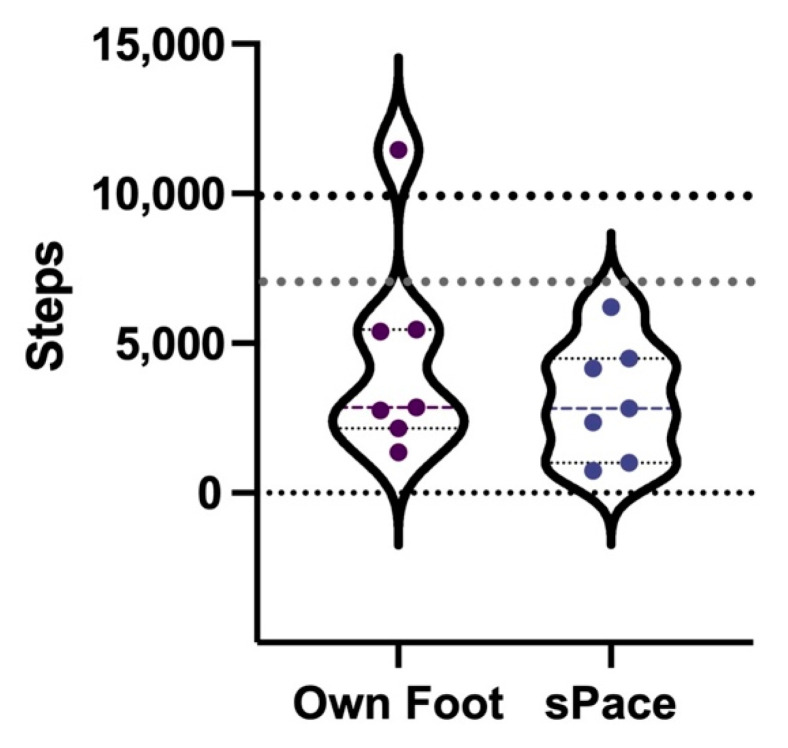
Average daily steps of participants wearing their own or sPace prosthetic foot (*n* = 7). Note: violin plots show distribution of data, dark dashed line indicates mean, lighter dotted lines indicate upper and lower quartiles, and gray and black horizontal dots indicate recommended daily step count ranges of 7000–10,000 steps.

Results of comparisons of physical activity in regard to pedometer step counts between the two prosthetic feet show no non-inferiority; however, no significant differences were observed *p* = 0.176. Users took on average 4490 ± 3444 steps in seven days when using the prosthesis with their own foot, compared to 3115 ± 1967 steps with the sPace foot (Figure 6). 

## 4. Discussion

In this study, participants completed performance based outcome measures to assess high-level mobility while using an sPace Vari-Flex foot. Moreover, habitual physical activity between users wearing sPace and their own foot was assessed. Results on the battery of performance-based measurements between feet vary, with the 10MWT showing comparable performances between the feet. Likewise, results of the 20MWT at CWS and FWS are comparable between feet; however, this was not seen in the uneven terrain condition. In the 2-MWT, non-inferiority was not seen, yet differences were still below the MDC of 34.3 m [26]. Results of the CHAMP battery showed non-inferiority between the study feet on the ESST, with our result being below the MDC of 1.53. Results of the IAT, *t*-test and SLS do not show non-inferiority; however, they were below the MDC of 1.10, 0.9 and 1.09, respectively [27]. Pearson correlations between the 2-MWT and CHAMP when participants wore either the sPace or Vari-Flex foot were both strongly positive. Lastly, habitual step counts between the two feet did not satisfy non-inferiority but had no significant differences. Taken together, these findings suggest a sustainable ESR foot option for prosthesis users residing in LMIC. 

In our participants, 10MWT speeds in either foot were faster and comparable to those reported in the literature for K3 ambulators (CWS 0.88 m/s and FWS 1.12 m/s) [19], (1.33 m/s) [28] and K4 ambulators (CWS 1.21 and 1.56 FWS) [19], respectively. Uneven terrain assessment is a relatively novel outcome measure in the lower limb prosthesis user population with limited published data on the parameter. Some scholars have integrated uneven foam pads into a novel uneven Figure-of-Eight Walk Test; however, this assessment involves a series of turns which make comparisons to our results challenging [28]. Still, the inclusion of an uneven terrain assessment in our study challenged participants and resulted in slower speeds than even those seen in terrain walking (Table 1). Uneven terrain is oftentimes a daily reality experienced by persons residing in LMICs such as SE Asia [29]. Although the 10MWT is a common test, we included the 20MWT as an additional challenge to explore performances between the two prosthetic feet. In this test, our participants’ speeds were on average 1.25 m/s for both prosthetic feet types at CWS, which is right above the speed at which a risk of all-cause mortality in knee osteoarthritic individuals is seen [30]. Again, as expected, uneven terrain challenged participants and resulted in slower speeds than even those seen in terrain trials. The 2-MWT is a widely utilized assessment of walking capacity in persons with varying disabilities [18]. The average distance covered in able-bodied persons is 199.1–209.7 m [31], whereas participants using the sPace and Vari-Flex feet covered 124.8 m and 123 m, respectively. In the literature, trans-tibial prosthesis users have reported distances of 143.8 ± 37.5 m and 154.1 ± 27.6 m [32,33]. As there is no ceiling effect in the lower limb prosthesis user population [34], it is an ideal measure for evaluating the effects of a prosthetic treatment and tracking progress towards rehabilitation goals over time. 

The CHAMP was initially designed to assess service members with traumatic limb loss, and an ability to perform the CHAMP requires a high level of mobility. According to the authors, mobility constitutes community ambulation and transitional movements, whereas high-level mobility places a demand on the musculoskeletal system to perform rapid speed movements in an efficient manner [27]. Thus, the CHAMP is distinctly different from the other outcome measures administered in this study. Results indicate that participants averaged a score of 16.94 ± 5.41 and 16.72 ± 6.09 with the sPace and Vari-Flex feet, respectively. These scores are lower than those observed in trans-tibial service members, at 26.85 ± 5.35; however, our participants’ scores may have been a result of the lower fitness levels of our study group. Correlation tests revealed strong positive Pearson correlations between the 2-MWT and CHAMP on both prosthetic feet. Recently, a correlation of *r* = 0.83 between 2-MWT and CHAMP was seen [21]. This result as well as ours supports the use of the CHAMP if limited in space and time [32].

The battery of tests used in the CHAMP are typically used for able-bodied persons. Still, as more groups of people with varying disabilities begin to participate in sports, valid measures of agility will be warranted. For example, the Illinois Agility Test (IAT) is a common assessor of agility in sports athletes [35]. As such, it was chosen as a means of assessing high mobility in our prosthesis-wearing participants. Likewise, the IAT is considered to be a useful measure of agility in wheelchair users [36]. Still, we did not perform aerobic capacity testing, which would have helped further elucidate high-level mobility and fitness level. Aerobic capacity testing, such as the maximal volume of oxygen uptake test (VO_2_ Max), is a reliable criterion measure of an individual’s aerobic capacity [37]. Running on a treadmill is not an easy task for prosthesis users; however, combined upper and lower limb ergometry [38] and arm ergometry [39] may be used instead if a criterion measure of aerobic capacity is needed.

Physical activity is an important lifestyle factor for ameliorating the negative health effects of disability [16,40]. Although activity was not increased when participants wore the sPace, they maintained their habitual activity levels observed with their own prosthetic foot. Participant step counts were lower than those of other trans-tibial prosthesis users at 5318 ± 2066 [41], and were well below the recommend activity for health (7000–10,000 steps per day) [42]. Still, these findings are important as they add to the body of literature on the objective measurement of activity in lower limb amputees [43]. More work is needed to explore facilitators and barriers to the promotion of activity in this population [44,45].

Our study had a number of observed limitations. The primary limitation was a low sample of high-functioning persons with traumatic limb loss. A broader, more heterogenous sample with subgroups based on amputation level, time since amputation and fitness would confirm or refute the ability to provide more generalizable findings for the prosthesis-wearing community. Our prosthetic foot accommodation time was short but if longer may have resulted in different participant performances. However, there is no established accommodation period for prosthetic feet. Some scholars have suggested as little as a few hours [16] to upwards of 4 weeks to ensure sufficient gait experience with a prosthetic foot [46,47]. No exclusion criteria were used with respect to participants’ everyday prostheses. Most participants used a SACH foot, but two used a single-axis foot. The differences in foot stiffness may have affected subjects’ walking symmetry when using the study feet. Likewise, prosthetic suspension was not controlled in this study. Suspension may alter prosthetic user performance. We recruited users with at least one year of prosthesis experience. However, recruiting new users or experienced users may offer a more generalizable report of performance in these prosthetic feet. Lastly, a comprehensive gait analysis is merited in order to provide possible underlying mechanisms of outcome measure performance. Self-report assessment of foot preference was not recorded. This subjective information is an important part of understanding user preferences. Lastly, the original CHAMP asks participants to complete three trials as opposed the two we asked our participants to perform out of an abundance of caution. Although the actual price is still being determined, the sPace foot is expected to be priced competitively and within reason for the Thai government universal healthcare scheme.

## 5. Conclusions

This study measured the performance-based outcome measures in trans-tibial prosthesis users wearing a local ESR prosthetic foot. There were no clear differences between the local and commercial prosthetic feet across measures of mobility and high-level mobility. The sPace foot performed well in a battery of high-level mobility outcome measures. Taken together, these data suggest the potential use of the sPace prosthetic foot for prosthesis users in LMICs. Moving forward, we will continue to investigate both free-living behavior as well as patient-reported outcomes across the continuum of prosthetic treatment for individuals using the sPace foot.

## Figures and Tables

**Table 1:** Results of performance-based outcome measures in study participants wearing sPace and Vari-Flex feet.

Tests	sPace	Vari-Flex	Mean Difference ± SD	Treatment Effect (95%CI)	*p*-Value
Walk Tests (m/s)					
10MWT Even Terrain					
CWS	1.25 ± 0.22	1.26 ± 0.18	0.01 ± 0.20	0.00 (−0.14–0.14)	0.015
FWS	1.66 ± 0.28	1.25 ± 0.45	0.40 ± 0.25	0.40 (0.18–0.61)	0.00042
10MWT Uneven Terrain					
CWS	1.14 ± 0.35	1.18 ± 0.34	0.03 ± 0.08	−0.03 (−0.08–0.02)	0.031
FWS	1.51 ± 0.46	1.56 ± 0.49	0.05 ± 0.10	−0.05 (−0.13–0.03)	0.099
20MWT Even Terrain					
CWS	1.28 ± 0.15	1.26 ± 0.09	0.01 ± 0.12	0.02 (−0.08–0.12)	0.013
FWS	1.68 ± 0.27	1.65 ± 0.20	0.02 ± 0.19	0.03 (−0.10–0.17)	0.03
20MWT Uneven Terrain					
CWS	0.87 ± 0.20	0.88 ± 0.22	0.008 ± 0.04	−0.008 (−0.06–0.05)	0.05
FWS	1.01 ± 0.23	1.05 ± 0.25	0.04 ± 0.08	−0.04 (−0.14–0.06)	0.10
2-MWT (m)	137.08 ± 40.50	138.90 ± 51.31	1.82 ± 23.05	−1.40 (−20.68–17.85)	0.33
CHAMP	16.94 ± 5.41	16.72 ± 6.09	0.22 ± 1.56	0.31 (−0.79–1.42)	0.116
Edgren Side-Step Test	2.77 ± 0.97	2.66 ± 1.11	0.11 ± 0.60	0.12 (−0.37–0.62)	0.049
Illinois Agility Test	5.14 ± 1.57	5.14 ± 2.03	0.00 ± 0.57	0.04 (−0.48–0.56)	0.097
*t*-test	6.00 ± 2.29	5.88 ± 2.02	0.11 ± 0.78	0.15 (−0.43–0.73)	0.12
Single-Limb Stance	5.35 ± 0.37	5.35 ± 0.55	0.00 ± 0.70	0.02 (−0.71–0.76)	0.18

Note: m/s: meters per second, m: meters, CIs: confidence intervals, SD: standard deviation, 10MWT: 10-m walk test, 20MWT: 20 m walk test, 2-MWT: two-minute walk test, CHAMP: Comprehensive High Activity Mobility Predictor.

## Data Availability

Not applicable.
